# Histopathological and immunohistochemical characterization of pulp tissue reaction to ACTIVA BioACTIVE base/liner in primary teeth pulpotomy: a randomized clinical trial

**DOI:** 10.1186/s12903-025-06177-x

**Published:** 2025-06-03

**Authors:** Shaimaa M. Mahfouz Omer, Heba Abdul Fattah Elsaied, Wafaa Yahia Alghonemy, Shaimaa S. EL-Desouky

**Affiliations:** 1 Pediatric Dentistry, Preventive Dentistry, and Dental Public Health Department, Faculty of Dentistry, Suez University, Suez, Egypt; 2 Oral Biology Department, Faculty of Dentistry, Suez Canal University, Ismailia, Egypt; 3 Oral Biology Department, Faculty of Dentistry, Sinai University- Kantara Branch, Ismailia, Egypt; 4 Basic Medical and Dental Sciences Department, Faculty of Dentistry, Zarqa University, Zarqa City, 13110 Jordan; 5 Oral Biology Department, Faculty of Dentistry, Tanta University, Tanta, Egypt; 6 Oral Health, and Preventive Dentistry Department, Faculty of Dentistry, Pediatric Dentistry, Tanta University, Tanta, Egypt

**Keywords:** Pulpotomy, MTA, ACTIVA BioACTIVE, Fibronectin, Osteopontin

## Abstract

**Background:**

Mineral trioxide aggregate (MTA) is the gold standard pulpotomy agent with some shortcomings, such as lengthy setting time, difficult manipulation, and a costly price. A new bioactive material, ACTIVA BioACTIVE Base/Liner, may prevail over these drawbacks.

**Aim:**

The purpose of the study was to evaluate and compare the pulp reaction in primary teeth to ACTIVA BioACTIVE Base/Liner and conventional powder-liquid MTA (WhiteProRoot®MTA) as pulpotomy agents.

**Materials and methods:**

Eighty primary first molars in children aged 7–9 years were assigned into two groups (40 molars/group) in which the pulpotomy procedure was done. The pulp tissue was dressed in ACTIVA BioACTIVE Base/Liner (group I) and MTA (group II). Twenty teeth from each group were extracted after 15 days, and the remaining twenty teeth in each group were extracted after 30 days. All teeth specimens underwent a decalcification treatment for histological and immunohistochemical assessment for fibronectin and osteopontin (OPN) expressions.

**Results:**

Significant statistical differences were identified between ACTIVA BioACTIVE and MTA groups concerning the total scoring of pulp vascularity, pulp fibrosis, and presence or absence of pulp stone (*P* = 0.015, *P* < 0.001, *P* = 0.038, respectively), whereas a non-significant difference was noted concerning the odontoblastic layer organization. Fibronectin and OPN in both ACTIVA BioACTIVE and MTA groups were positive at the fibrotic and calcified areas.

**Conclusion:**

ACTIVA BioACTIVE Base/Liner demonstrated encouraging outcomes concerning enhanced systemic tissue responses.

**Trial Registration:**

ClinicalTrials.gov, NCT05300152, “Pulpotomy Medications in Primary Teeth”, Registered: 29/03/2022, https://clinicaltrials.gov/study/NCT05300152?cond=NCT05300152&rank=1

## Introduction

Dental caries is still one of the most common health issues among children worldwide, particularly in developing nations. One of the most challenging problems that a pediatric dentist faces today is the treatment of deep caries in primary teeth. Because of the long-term existence of these lesions, a high percentage of them are linked to pulpal exposure [1]. Due to the low success outcome of direct pulp capping in primary teeth, a vital pulpotomy is an acceptable and widely used procedure to treat carious exposures of asymptomatic vital primary teeth [2].

A pulpotomy is an endodontic technique wherein the coronal pulp tissue is removed, and the residual root pulp is covered with a bactericidal, biological barrier-forming pulp medicament [3]. Formocresol is the standard capping agent for pulpotomy of primary teeth due to its higher clinical performance and ease of application [4]. On the other hand, toxic effects, genetic mutation, and carcinogenicity risks presented by the likely systemic diffusion of formocresol molecules through the root canals aroused reservations regarding their application [5]; Consequently, a safer, more biocompatible medication was needed.

The use of calcium silicate-containing bioactive materials in pediatric dentistry has increased recently due to their favorable characteristics including stimulating the regeneration of pulp cells, shifting the inflammatory response, and enhancing the healing ability of the residual vital pulp [6]. MTA represents the new benchmark for pulp capping therapy [7]; it is a biocompatible material that seals more effectively than zinc oxide eugenol [8]. Furthermore, it preserves pulp vitality and encourages repair as it encounters dental pulp or peri-radicular tissues [9]. However, MTA has some disadvantages including difficulty handling, high prices, lengthy setting time, weak mechanical characteristics, weak adhesion to dental tissue, and tooth discoloration [10].

ACTIVA BioACTIVE Base/Liner is a light-cured resin-modified calcium silicate (RMCS) that integrates key features of both composite resins and glass ionomers [11]. It is made from diurethane and methacrylate-based monomers, combined with a modified polyacrylic acid and a rubber-reinforced diurethane dimethacrylate, with BioACTIVE glass used as the filler [12]. Compared to glass ionomers, it offers enhanced release and recharge of calcium, phosphate, and fluoride, all within a durable resin matrix that resists chipping and crumbling. It also promotes hydroxyapatite production and remineralization at the material-tooth interface [13]. ACTIVA BioACTIVE base/liner is free from Bisphenol A (BPA), Bis-GMA, and BPA-related compounds and also adheres directly to dentin without the need for etching or bonding agents [13]. Although the manufacturer does not currently list vital pulp therapy, such as direct pulp capping or pulpotomy, as an indication, emerging studies have suggested a possible role for ACTIVA BioACTIVE Base/Liner in such procedures due to its bioactivity and biocompatibility [13, 14]. Kunert et al. [11], reviewed available data and acknowledged that the use of ACTIVA BioACTIVE Base/Liner in vital pulp therapies might be justified based on its composition, while also cautioning that the resin components could potentially influence the pulp’s inflammatory response and require further investigation through in vitro and in vivo models. In contrast, Abou ElReash et al., [14] reported that ACTIVA BioACTIVE exhibited excellent biocompatibility and healing capacity in rat subcutaneous tissue, comparing favorably with traditional calcium silicate-based materials. Moreover, a study carried out by Karabulut et al., [13] found that the histological response to ACTIVA BioACTIVE Base/Liner was very similar to Biodentine® and ProRoot MTA, with all three materials showing good tissue tolerance over a 60-day evaluation period. Notably, dystrophic calcification was observed in the connective tissue adjacent to ACTIVA, suggesting reparative activity and further supporting its potential as a pulp capping material. These findings highlight the potential of ACTIVA BioACTIVE as an alternative bioactive material for vital pulp procedures and support the need for further histological and immunohistochemical evaluations, particularly in the context of primary teeth pulpotomy.

There is inadequate evidence to precisely evaluate ACTIVA BioACTIVE’s reparative ability and its impact on the human vital pulp; therefore, the purpose of this study was to assess and compare the pulp response in primary teeth to ACTIVA BioACTIVE Base/Liner and MTA as pulpotomy medications. The null hypothesis (H_0_) assumed no difference in pulp response after primary teeth pulpotomy using ACTIVA BioACTIVE Base/Liner and MTA after 15- and 30-day intervals.

## Materials and methods

### Study setting and ethical considerations

A randomized controlled prospective clinical trial was carried out in the Outpatient Clinics of the Pediatric Dentistry Department at the Faculty of Dentistry, Suez Canal University, from October 2022 to December 2023. This study adheres to the CONSORT guidelines. The trial was registered with the identifier NCT05300152 at ClinicalTrials.gov (Registered: 29/03/2022). The ethical committee (REC) of the Faculty of Dentistry, Suez Canal University provided ethical permission for this study, with code (398/2021) following the Helsinki Declaration of 1964 and its later revisions. Once parents signed a written informed consent, clinical treatment (pulpotomy procedure) was started. Then, the pulpotomized teeth were extracted after 15-day and 30-day intervals for histological evaluation and immunohistochemical assessment of Fibronectin/Osteopontin expression.

### Eligibility criteria

**Fig. 1:**
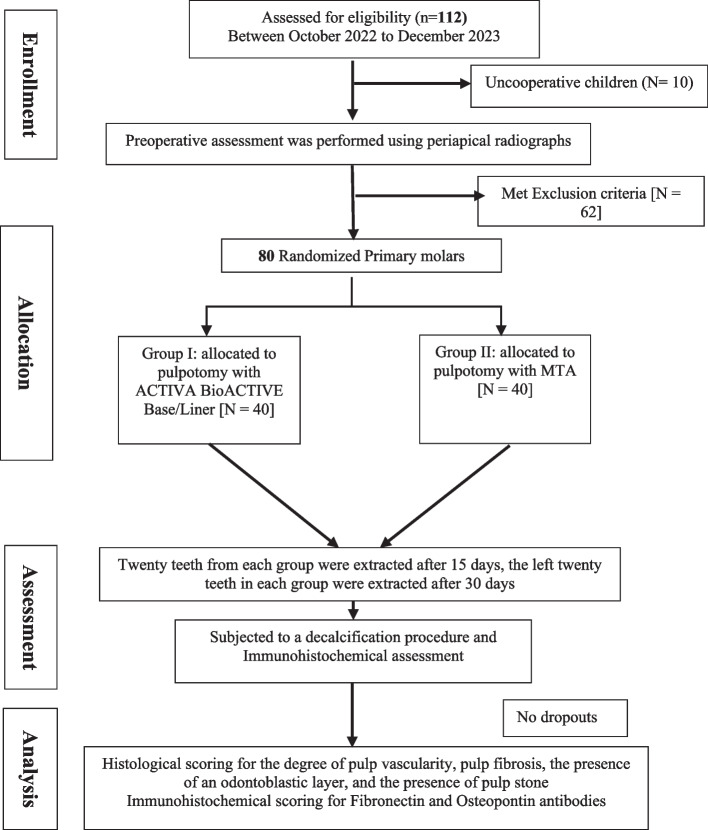
Flowchart outlining the study's randomization and allocation

A total of 112 child patients aged 7–9 years were enrolled and assessed to meet the inclusion and exclusion criteria of the present study. Inclusion criteria were apparently healthy cooperative children with bilateral primary first molars that require dental treatment owing to occlusal and/or proximal deep caries (ICDAS code 5 or 6) [15] and need orthodontic serial extraction. The selected primary molars should have at least an intact two-thirds of the root (confirmed by pre-operative periapical x-ray). Children with a history of short-lasting, localized pain that subsided following the removal of the triggering mechanical or thermal stimulus, or children who had experienced a recent history of spontaneous pain of momentary duration within the previous seven days, were included [16]. Children with a history of irreversible pulpitis symptoms and signs, including percussion sensitivity, spontaneous pain, fistulous tract associated with the affected tooth, pathologic tooth mobility, and inter-radicular radiolucency, were excluded. Also, children with systemic diseases and uncooperative patients were eliminated from this study. Accordingly, 72 child patients were precluded, leaving the final study sample of forty children. Enrollment, allocation, assessment, and sample size analysis are displayed in a flow chart in Fig. 1.

### Sample size calculation & randomization

The sample size calculation was performed utilizing G Power version 3.1.9.2. The effect size conventions (d) were 0.75 (large) based on the mean difference of the sample size calculation of Mohamed et al., [17] using Beta (β) level of 0.05 and alpha (α) level of 0.05, i.e., power = 95%; the calculated sample size (n) as a minimum should be 80 samples and will be divided equally for two groups (40 samples each).

The Research Randomizer program was used to carry out the randomization procedure (https://www.randomizer.org) [18] An autonomous individual produced a computer-created random list enclosed in a wrapped envelope and assigned those who fulfilled the inclusion criteria to one of the two groups.

### Group assignment

Eighty primary first molars were allocated into two groups (40 molars/group) randomly according to different pulpotomy agents:

- Group I (experimental group) (*n* = 40): primary first molars were treated with ACTIVA BioACTIVE Base/Liner (Pulpdent, Watertown, MA, USA).
- Group II (positive control group) (*n* = 40): primary first molars were treated with the conventional powder-liquid Mineral trioxide aggregate (MTA) (WhiteProRoot®MTA, Dentsply Sirona, York, PA, USA).

### Pulpotomy procedure

A single operator carried out a pulpotomy procedure under strictly aseptic conditions. Before initiating the pulpotomy treatment, a perioperative assessment was performed utilizing an intraoral digital photo-simulated phosphorus plate sensor (Planmeca ProSensor HD, Helsinki, Finland). The teeth received anesthesia with 2% mepivacaine with 1:20,000 levonordefrin(Alexandria Co.), then isolated with a rubber dam (Midwest Dental, Texas, USA). Caries removal was initially performed using sterile No. 330 carbide burs in a high-speed contra-angle handpiece under copious water cooling, avoiding entry into the pulp chamber. The remaining carious dentin was removed using a spoon excavator or a slow-speed round bur as needed. Access to the pulp chamber was achieved by removing its roof with a slow-speed round bur [19]. Coronal pulp tissue was then excised using a large excavator or large round bur, followed by irrigation of the chamber with sterile saline solution. Clinically, the exposed pulp tissue was assessed; it should be red and vital, without any grey discoloration that may suggest abscess formation or necrosis [20]. Also, hemostasis was achieved by gently applying a moistened sterile cotton pellet with light pressure for a duration of 3 to 5 min [16]. The tooth was precluded if the bleeding had not ceased in 5–10 min, and pulpectomy was indicated [20]. Then, pulpotomy agents were utilized based on each group. In group I (*n* = 40): the pulp tissue was dressed with ACTIVA BioACTIVE Base/Liner in a thickness of about 0.5 mm, which was delivered via an auto-mix syringe and then, as directed by the manufacturer, light-cured for 20 s [21]. While in group II (*n* = 40), the pulp tissue was topped with conventional powder-liquid MTA in a thickness of about 1.5–2 mm [22]. The MTA paste was done by mixing 0.16 g MTA powder with sterile saline to produce a uniform paste [23]. Then, a reinforced zinc oxide-eugenol layer (IRM® DENTSPLY International, USA) was applied [24] preceding glass ionomer cement filling (Medfill Promedica, Germany).

Children were recalled after 15- and 30-day time intervals. Twenty teeth of group I and twenty of group II were extracted after 15 days and then exposed to a decalcification procedure. Later, the left twenty teeth in every group were extracted after 30 days and underwent a decalcification procedure.

### Tissue preparation

Teeth were immediately preserved in 10% formalin for a minimum of 10 days after extraction and then subsequently submerged in a 10% ethylenediaminetetraacetic acid (EDTA) solution for around 4 weeks till fully decalcified [1]. Then, the specimens underwent routine processing and were encased in paraffin wax. [25]. Labiolingual longitudinal successive sections were cut on a microtome at 5 µm thickness, and each section was de-paraffinized with xylene [26] and re-hydrated in descending alcohol concentrations. Finally, each section was briefly cleaned in distilled water before being stained for 8 min in Harris hematoxylin solution.

The eosin-phloxine solution was used to counterstain. Finally, Sections were mounted using a xylene-based mounting solution for standard histological examination under a light microscope (Leica ICC50 HD) with two well-trained oral histopathologists. Throughout the study, coded samples were adopted to prevent potential bias.

The histological assessment was performed based on the scoring standards of Heyeraas et al., [27] and Omar et al., [28] as follows: the degree of pulp vascularity (1: mild, 2: moderate, 3: severe), pulp fibrosis (0: no fibrosis, 1: mild, 2: moderate, 3: severe), the presence of an odontoblastic layer (0: organized, 1: non-organized) and the presence of pulp stone (0: no pulp stone, 1: the presence of pulp stone).

### Immunohistochemical assessment of Fibronectin expression [29]

Firstly, the sections were heated for one hour at 65 °C in an oven. Then, de-paraffinization/hydration was done using two xylene washes, two rinses of 100% ethanol, and descending alcohol concentrations, and finally, rinsing with water and a Tris–HCl Buffered Saline (TBST). After that, the sections were soaked in a staining dish with an Antigen Retrieval solution and put in a rice cooker for about 20 to 30 min. Then, they were allowed to cool for 20 min. Concerning section staining, it was rinsed with TBST and coated with 3% hydrogen peroxide to deactivate the endogenous peroxidase. Then, they were washed thrice with TBST and blocked with the blocking solution. The primary antibody (anti-human fibronectin polyclonal antibodies) was diluted per the manufacturer's recommendation, then applied to each section, and stored overnight in the humidified chamber (4 ℃) [30]. A secondary horseradish peroxidase (HRP)-conjugated anti-rabbit antibody diluted in the blocking solution was put on following the manufacturer's recommendations and incubated at room temperature for 1 h. The sections were rinsed thrice (3 min each on a shaker) with TBST before and after the secondary antibody application. A freshly produced diaminobenzidine (DAB) substrate was put on the sections and incubated till the desired staining developed (2 to 5 min) at room temperature. Finally, sections were rinsed with water and counterstained with Hematoxylin.

### Immunohistochemical assessment of Osteopontin (OPN) expression [31]

To identify OPN synthesis, the immunohistochemical assessment was done via the avidin–biotin-peroxidase complex (ABC) technique with a VECTASTAIN ABC Kit (Vector Laboratories Inc., Burlingame, California, USA). After deparaffinization, sections were subjected to Proteinase-K treatment (Roche Diagnostics, Mannheim, Germany) and subsequently incubated with primary antibody (Rabbit polyclonal anti-osteopontin (LSL Co., Ltd, Tokyo, Japan)). Then, the sections were incubated with biotinylated IgG, streptavidin–horseradish peroxidase, and a substrate of 3,3′-diaminobenzidine (Funakoshi Co., Ltd, Tokyo, Japan). A negative control was non-immune serum. To detect the osteoclasts and mononuclear precursors, tartrate-resistant acid phosphatase (TRAP) staining was performed using a TRAP staining kit (Hokudo Corp., Sapporo, Japan). In the connective tissue next to the material, OPN was detected as brown color areas. The positive reaction area in images captured with a high-resolution camera (Carl Zeiss Imager D1 Axio, Goettingen, Germany) connected to an ocular microscope (Axio Cam MRc5; Carl Zeiss Microimaging, Thornwood, NY) (× 20) was measured via the image analyzer program (Leica QWin software, version 3.7.0, Switzerland).

### Statistical analysis

The SPSS software, version 26.0 for Windows, was used to analyze all gathered data. Cohen's kappa was employed to assess the inter-examiner agreement. A normality test (Shapiro–Wilk) was performed to ensure the samples had a normal distribution. Descriptive statistics were derived as mean ± SD. The different groups were compared using an independent sample T-test. The chi-square test of independence was used to compare nominal or categorical variables. *P*-value < 0.05 indicates statistical significance.

## Results

A total of eighty pulpotomy procedures were performed in children ranging from seven to nine years. The age distribution showed that the majority of participants in both groups were 9 years old (50% in Group I and 55% in Group II), with a mean age of 8.25 ± 0.84 years in the ACTIVA BioACTIVE group and 8.38 ± 0.77 years in the MTA group with no significant difference (*P* = 0.932). Male children comprised 45% of Group I and 55% of Group II, while females were 47.5% and 52.5%, respectively, with no statistically significant differences (*P* = 0.8225) (Table 1). All patients were presented for 15- and 30-day time intervals. Inter-examiner reliability, evaluated using Cohen’s kappa (κ = 1.00) for all variables, reflected almost perfect agreement at 15 days. Cohen's Kappa was used to estimate inter-rater reliability rather than simple percent agreement because it accounts for the possibility of random agreement [32]. Kappa statistics were interpreted as following: (0 or less than 0) denotes poor agreement, (0.01—0.20) denotes slight agreement, (0.21—0.40) denotes fair agreement, (0.41—0.60) denotes moderate agreement, (0.61—0.80) denotes substantial agreement, and (0.81—1.00) denotes almost perfect agreement. In this study, Cohen’s kappa was more than 0.81, indicating almost perfect agreement between the two examiners as presented in Table 2.

**Table 1:** Demographic distribution of study sample

Demographic characteristics	Group-I (ACTIVA BioACTIVE) (N = 40)	Group-II (MTA) (N = 40)	Test	*P*-value
Age in years				
7	10 (25%)	7 (17.5%)	0.588^a^	0.744
8	10 (25%)	11(27.5%)		
9	20 (50%)	21(55%)		
Mean age	8.25 ± 0.84	8.38 ± 0.77	1.35 ^b^	0.932
Gender				
Male	18 (45%)	22(55%)	0.050 ^a^	0.8225
Female	19 (47.5%)	21 (52.5%)		
a; chi square test b; independent samples T test

**Table 2:** Interpretation of Cohen’s kappa at a 15-day interval for both groups

Histopathological criteria	Scoring	Group-I (ACTIVA BioACTIVE) (N = 20)		Cohen’s Kappa	Group-II (MTA) (N = 20)		Cohen’s Kappa
		**Examiner** **(1)**	**Examiner** **(2)**		**Examiner** **(1)**	**Examiner** **(2)**	
Pulp vascularity	1 (mild)	14	13	0.96	12	11	0.96
	2 (moderate)	6	7		4	5	
	3 (severe)	-	-		4	4	
Pulp fibrosis	0 (No fibrosis)	-	-	1.00	-		1.00
	1 (Mild fibrosis)	-	-		16	16	
	2 (Moderate fibrosis)	18	18		4	4	
	3 (Severe fibrosis)	2	2		-	-	
Odontoblastic layer	0 (Organized)	4	4	1.00	2	3	0.96
	1(Non-organized)	16	16		18	17	
Pulp stones	0 (No pulp stone)	16	16	1.00	20	20	1.00
	1 (Presence of pulp stone)	4	4		0	0	

### Histopathological results

**Fig. 2:**
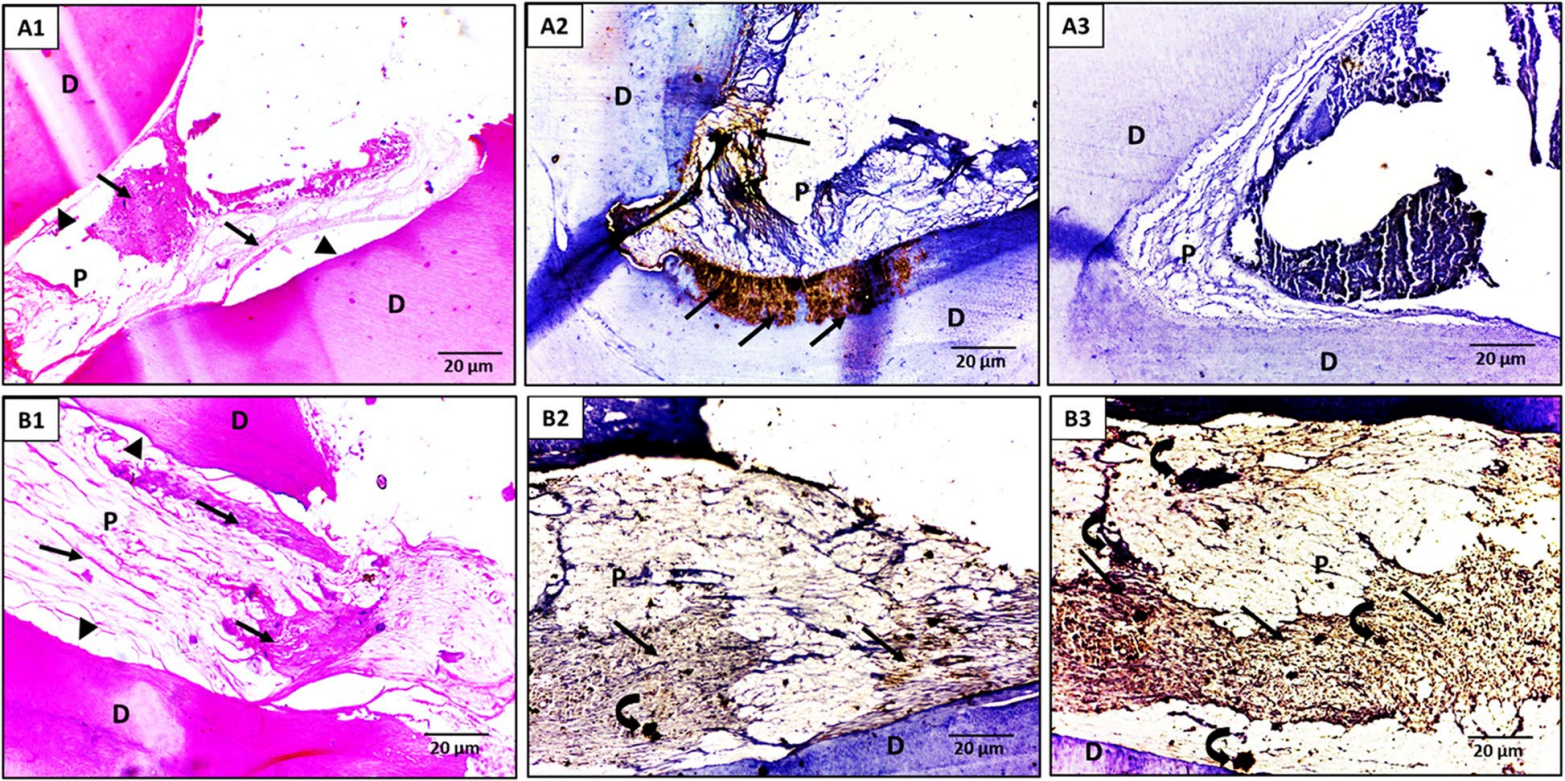
A light micrograph of the ACTIVA BioACTIVE group. A1-A3; after 15 days post-treatment interval, (A1) The pulp tissue showed pulp fibrosis (arrows) and loss of odontoblastic layer (arrowheads), (A2) Immunohistochemical localization of fibronectin (arrows) was slightly detected in the pulp fibrosis and strongly noticed in the dentin layer at the pulp periphery, (A3) Immunohistochemical localization of OPN was nearly negative expressed on the pulp and dentin. (B1-B2; after 30 days post-treatment interval, (B1) The pulp tissue showed diffuse pulp fibrosis, nearly occupying the pulp space (arrows), and loss of odontoblastic layer (arrowheads), (B2) Immunohistochemical localization of fibronectin was strongly detected in the pulp matrix and the areas of pulp fibrosis (arrows), as well as in a small, calcified pulp stone (curved arrow), (B3) Immunohistochemical localization of OPN was strongly detected in the pulp matrix and the areas of pulp fibrosis (arrows) as well as in multiple calcified pulp stones (curved arrows). (D) Dentin, (P) pulp. (Orig. Mag.X400)

**Fig. 3:**
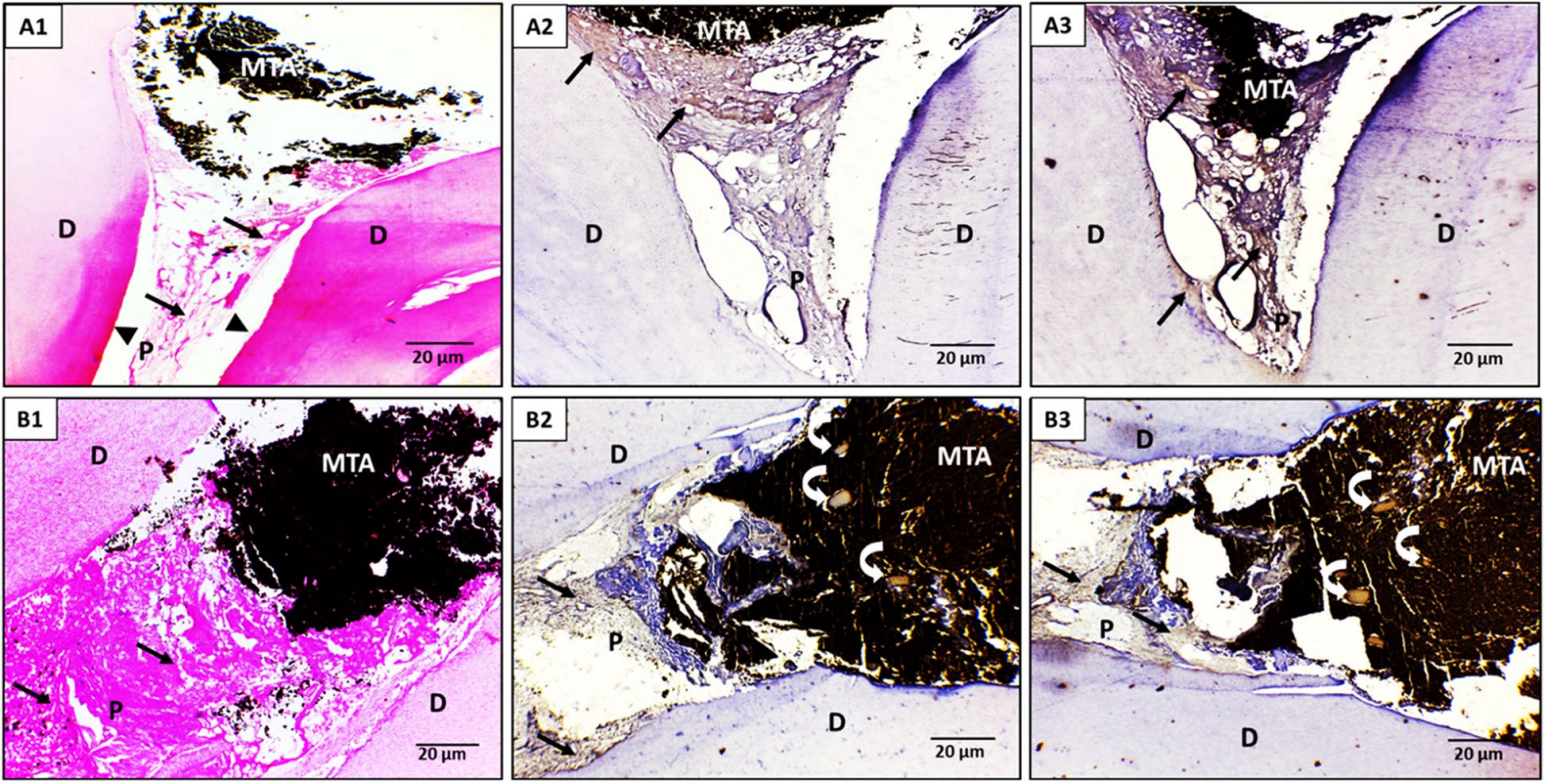
A light micrograph of the MTA group. A1-A3; after 15 days post-treatment interval, (A1) The pulp tissue showed pulp fibrosis (arrows) and loss of odontoblastic layer (arrowheads), (A2) Immunohistochemical localization of fibronectin (arrows) was moderately detected in the pulp fibrosis, (A3) Immunohistochemical localization of OPN (arrows) was moderately noticed in the pulp fibrosis and in the dentin layer at the pulp periphery. B1-B3; after 30 days post-treatment interval, (B1) The pulp tissue showed increased pulp fibrosis (arrows) occupying all the pulp space, (B2) Immunohistochemical localization of fibronectin (arrows) was moderately detected in the pulp fibrosis (straight arrows) and in some calcified pulp stones (curved arrows), (B3) Immunohistochemical localization of OPN (arrows) was moderately noticed in the pulp fibrosis (straight arrows) and strongly detected in some calcified pulp stones (curved arrows). (D) Dentin, (P) pulp. (Orig. Mag. X400)

Concerning the pulp vascularity, at a 15-day interval, both ACTIVA BioACTIVE and MTA groups exhibited predominantly mild to moderate vascular changes, with ACTIVA showing 70% mild and 30% moderate alterations versus MTA’s 60% mild, 20% moderate, and 20% severe, with no statistically significant difference (p = 0.320) ​. By a 30-day interval, however, ACTIVA BioACTIVE shifted toward a healthier vascular profile (50% mild, 50% moderate), whereas MTA still displayed 40% severe pulp vascularity alongside 50% moderate and only 10% mild changes, with a statistically significant difference (p = 0.0356) (Table 3, Figures-2, 3).

**Table 3:** The score percentages for the studied groups regarding the evaluated histopathological criteria at both follow-up intervals

Histopathological criteria	Scoring	Group-I (ACTIVA BioACTIVE) (N = 40)		Group-II (MTA) (N = 40)		P1-value	P2-value
		**15-days**	**30-days**	**15-days**	**30-days**		
Pulp vascularity	1 (mild)	14(70%)	10(50%)	12(60%)	2(10%)	0.320	0.0356*
	2 (moderate)	6(30%)	10(50%)	4(20%)	10(50%)		
	3 (severe)	-	-	4(20%)	8(40%)		
*Chi-square P-value*		0.361		0.063			
Pulp fibrosis	0 (No fibrosis)	-	-	-	-	0.0011*	0.021*
	1 (Mild fibrosis)	-	-	16(80%)	4(20%)		
	2 (Moderate fibrosis)	18(90%)	10(50%)	4(20%)	16(80%)		
	3 (Severe fibrosis)	2(10%)	10(50%)	-	-		
*Chi-square P-value*		0.050*		0.007*			
Odontoblastic layer	0 (Organized)	4(20%)	4(20%)	2(10%)	2(10%)	0.531	0.531
	1(Non-organized)	16(80%)	16(80%)	18(90%)	18(90%)		
*Chi-square P-value*		1.0		1.0			
Pulp stones	0 (No pulp stone)	16(80%)	12(60%)	20(100%)	18(90%)	0.136	0.121
	1 (Presence of pulp stone)	4(20%)	8(40%)	-	2(10%)		
*Chi-square P-value*		0.329		0.304			

^*^Significant difference at p value ≤ 0.05

P_1_-value: pairwise between group I & group II at a 15-days interval

P_2_-value: pairwise between group I & group II at a 30-days interval

Regarding pulp fibrosis, at a 15-day interval, ACTIVA BioACTIVE induced predominantly moderate to severe fibrosis; 90% and 10% of the samples exhibited moderate and severe pulp fibrosis, respectively. whereas MTA samples showed mostly mild (80%) and some moderate (20%) fibrosis with a statistically significant difference (*p* = 0.0011)​. By a 30-day interval, ACTIVA BioACTIVE maintained a robust fibrotic response with equal proportions of moderate and severe fibrosis (50% each), while MTA remained largely in the mild-to-moderate range (20% mild, 80% moderate), with a significant difference (*p* = 0.021) ​(Table-3, Figs. 2 and 3).

Concerning odontoblastic layer organization, at both 15-day and 30-day intervals, 20% of ACTIVA BioACTIVE samples displayed an organized odontoblastic layer, while the remaining 80% remained non-organized, with no significant difference between both groups at either interval (*p* = 0.531)​ (Table-3, Figs. 2 and 3).

Throughout the follow-up periods, ACTIVA BioACTIVE tended to induce more pulp‐stone formation than MTA, although none of these differences reached statistical significance. At a 15-day interval, 20% of ACTIVA BioACTIVE samples exhibited pulp stones versus 0% in the MTA group (*p* = 0.136), and by a 30-day interval, the incidence had increased to 40% for ACTIVA compared to 10% for MTA (p = 0.121). ​

As revealed in Table 4, ACTIVA BioACTIVE samples demonstrated significantly milder vascular changes, with a mean vascularity score of (1.40 ± 0.50) versus (1.95 ± 0.83) for MTA (*p* = 0.015)​. Also, ACTIVA BioACTIVE induced a stronger fibrotic response, averaging (2.30 ± 0.47) compared to (1.50 ± 0.51) in the MTA group (*p* < 0.001)​. There was no significant difference in odontoblastic layer organization between ACTIVA BioACTIVE (0.80 ± 0.41) and MTA (0.90 ± 0.31) (*p* = 0.389). Finally, ACTIVA BioACTIVE samples exhibited more pulp-stone formation (0.30 ± 0.47) versus (0.05 ± 0.22) for MTA (*p* = 0.038), indicating a modest but statistically significant increase in calcific deposits. ​

**Table 4:** Comparison of the mean total scoring for the studied groups regarding the evaluated histopathological criteria

Histopathological criteria	Groups	Range	Mean ± SD	T-test	*P*-value
Pulp vascularity	Group-I (ACTIVA BioACTIVE)	1–2	1.400 ± 0.503	2.545	0.015*
	Group-II (MTA)	1–3	1.950 ± 0.826		
Pulp fibrosis	Group-I (ACTIVA BioACTIVE)	2–3	2.300 ± 0.470	5.141	< 0.001*
	Group-II (MTA)	1–2	1.500 ± 0.513		
Odontoblastic layer	Group-I (ACTIVA BioACTIVE)	0–1	0.800 ± 0.410	0.872	**0.389**
	Group-II (MTA)	0–1	0.900 ± 0.308		
Pulp stones	Group-I (ACTIVA BioACTIVE)	0–1	0.300 ± 0.470	2.147	0.038*
	Group-II (MTA)	0–1	0.050 ± 0.224		

^*^Significant difference at *p* value ≤ 0.05

### Immunohistochemical results

Fibronectin and OPN in both ACTIVA BioACTIVE and MTA groups were positive at the fibrotic and calcified areas. Only Fibronectin was noticed at the newly formed dentin layer at the 15-day interval of ACTIVA BioACTIVE treatment (Figs. 2, 3).

As revealed in Table 5, significant statistical differences were reported between ACTIVA BioACTIVE and MTA groups at the 15-day (P = 0.002) and 30-day intervals (P = 0.008) regarding the presence of fibronectin. The fibronectin percent change from 15-day to 30-day intervals was increased significantly, with 26.01% in the ACTIVA BioACTIVE group compared to 9.11% in the MTA group. Concerning osteopontin levels, the ACTIVA BioACTIVE group exhibited an increase in OPN levels from (50.42 ± 4.81) on day 15 to (92.56 ± 4.06) at day 30 (p = 0.020), whereas MTA showed a smaller shift from (63.72 ± 2.91) to (75.55 ± 6.70) (p = 0.010). The percentage change in osteopontin was also significantly higher with ACTIVA BioACTIVE (83.58%) versus (18.57%) for MTA, indicating that ACTIVA BioACTIVE more robustly enhances osteopontin expression over the follow-up intervals (Table 5).

**Table 5:** Comparison of the study groups'mean & standard deviation (SD) for the levels of osteopontin and fibronectin at both follow-up periods

		**Group-I (ACTIVA BioACTIVE)** **(**Mean ± SD**)**		**Group-II (MTA)** (Mean ± SD)	**T-test**	***P*-value**
**Fibronectin**	15-day		58.52 ± 4.62	81.88 ± 3.13	**7.24**	0.002*
	30-day		73.74 ± 3.17	89.34 ± 4.55	**4.865**	0.008*
	% change		26.01	9.11		
	T-test		5.13	3.69		
	P-value		0.03*	0.231		
**Osteopontin**	15-day		50.42 ± 4.81	63.72 ± 2.91	**4.56**	0.010*
	30-day		92.56 ± 4.06	75.55 ± 6.70	**3.75**	0.020*
	% change		83.58	18.57		
	T-test		9.37	4.84		
	*P*-value		0.011*	0.04*		

^*^Significant difference at *p* value ≤ 0.05

## Discussion

MTA is commonly utilized in pulpotomies and exhibits great clinical and radiological success rates [23]. Also, it induces more homogenous, thicker dentin bridges in histological analysis [33]. On the other hand, its significant challenge for usage in paediatric dentistry refers to the expense of the material, taking into consideration its restricted use in developing countries and the existence time of the primary tooth in the oral cavity [34]. Also, it has a very lengthy setting time that may lead to the possibility of partial material loss and interface change during the finishing phase of the procedure [35]. Recently, ACTIVA BioACTIVE Base/Liner was introduced with the possibility of stimulating biomineralization, as MTA and Biodentine [36]. In addition, it has a favourable setting time that three-setting processes can explain without delay in applying the final restoration since it sets for 20 s with low intensity light for each layer and possesses both composite self-cure and glass-ionomer (acid–base) setting reactions. [12, 37]. Nonetheless, there is still inadequate evidence about its influence on the vital pulp and the reparative potential, so this research was targeted to assess and compare the histological and immunohistochemical analyses of pulp profiles after vital pulpotomy with ACTIVA BioACTIVE Base/Liner and MTA in primary teeth.

The current study's findings rejected the null hypothesis, as there were statistically significant differences between the two groups concerning the total scoring of pulp vascularity, pulp fibrosis, and the presence or absence of pulp stone. A non-significant difference was reported regarding the odontoblastic layer organization (the null hypothesis was partially accepted).

Regarding the pulp vascularity, a significant difference was found between ACTIVA BioACTIVE and MTA groups at the end of the 30-day interval with severe pulp vascularity detected in the MTA group; the inflammatory reaction of MTA may be likely stemed from local pH shifts, the heat released as it sets, and the ensuing production of pro-inflammatory cytokines such as IL-1 and IL-6 [38]. Also, shifting toward a healthier vascular profile of the ACTIVA BioACTIVE group (50% mild, 50% moderate) may be attributed to the lack of Bis-GMA in ACTIVA BioACTIVE's formulation, which could be a basis for its minimal genotoxicity and cytotoxicity [37]. This agreed with Louwakul and Lertchirakarn., [39], who observed acute inflammation in 80%, 60%, and 40% of Dycal, MTA, and pulp-capping material incorporating fluocinolone acetonide (PCFA), respectively, eight days after capping inflamed dental pulps of rat maxillary molars, while the MTA group reported slight to moderate inflammation after 30 days. Also, these results coincided with Salako et al., [40], who found localized areas of inflammation after two weeks of bioactive glass application as a pulpotomy agent in rat molars, while at four weeks, inflammation resolution occurred, and most of the slides demonstrated normal pulp tissue. Moreover, this was in line with Darweesh et al., [41], who revealed that after three weeks of subcutaneous implantation into Albino male rats, ACTIVA showed clear healing with inflammation having dropped markedly, and a dense fibrous capsule of parallel collagen fibers had formed. While in the same study, the MTA’s inflammatory response, though it lessened over time, remained mildly present at week three, with a loosely organized fibrous capsule and more inflammatory cells than seen with ACTIVA. Conversely, the current study results disagreed with Haghgoo & Ahmadvand., [42], who evaluated the pulpal response after direct pulp capping of primary teeth and observed inflammatory changes in one case in the MTA group compared to three cases in the bioactive glass group.

Regarding pulp fibrosis after a 30-day interval, 20% and 80% of the MTA group were recorded with mild and moderate pulp fibrosis, respectively; these results were comparable to those of Alzoubi et al. [43], study in which 40% of White MTA specimens presented no fibrosis and 60% had mild fibrosis after the 3rd month of follow-up. Moreover, in the ACTIVA BioACTIVE group, after the 30-day interval, moderate and severe pulp fibrosis were equally noticed (50%) in this study; this may be attributed to less adverse pulpal effects, non-evident heat generation, and reduced levels of ACTIVA BioACTIVE's unpolymerized poisonous monomers [44]. This aligns with the findings of Vouzara et al., [45], who reported the release of methacryloyloxyethyl phosphorylcholine (MOPA) from ACTIVA BioACTIVE base/liner, and their chromatographic analysis did not reveal the presence of Bisphenol A (BPA) or Bis-GMA, indicating that the material is formulated to reduce oxidative stress and probable cytotoxicity. Furthermore, Schneider et al. [46] demonstrated that Bis-GMA depletes glutathione and inhibits cystine uptake, which contributes to oxidative stress and subsequent cell death. Moreover, Kunert et al., [37] verified favorable biocompatibility and bioactive properties of the ACTIVA BioACTIVE liner; also, the ACTIVA BioACTIVE, Biodentine, and Predicta Bioactive revealed no substantial rises in apoptosis in the studied cell line, despite ProRoot MTA and MTA Angelus exhibiting some toxicity.

In the present study, no statistically significant differences were observed between ACTIVA BioACTIVE and MTA groups regarding odontoblastic layer organization at both follow-up intervals, with 20% of ACTIVA samples exhibiting organized odontoblast layers compared to 10% in the MTA group. This marginal difference suggests that while both materials support reparative activity, ACTIVA may offer a slight advantage in promoting early odontoblastic organization. This was consistent with Louwakul and Lertchirakarn., [39], who observed higher hard tissue formation rates with Dycal (78%) and pulp-capping material containing fluocinolone acetonide (PCFA) (100%) compared to MTA(63%), suggesting that certain bioactive formulations may exert a stronger inductive effect on dentinogenesis than MTA. Conversely, our findings contrasted with those of Haghgoo and Ahmadvand., [42], who reported more frequent dentin bridge formation with MTA than with bioactive glass (BAG) after direct pulp capping of primary teeth, suggesting variability in the performance of bioactive materials depending on their composition and interaction with pulp tissues. The unorganized odontoblast cells increased in the MTA group compared to the ACTIVA BioACTIVE group in this study; this corresponded to Shi et al., [47], who noted that connective tissue in the dentine bridge was not detected in the iRoot BP Plus group compared to its presence in one MTA specimen. The existence of connective tissue within the dentin bridge demonstrates that the bridges are not fully calcified [48]. This could be explained by the reparative dentine quickly forming in an unorganized manner and engulfing the cellular inclusions. Also, as the bridge matures and tubular dentine development starts, the reparative dentine may eventually become more regular and mineralized [49].

Non-significant differences were reported between ACTIVA BioACTIVE and MTA groups at both intervals regarding the presence of pulp stones, with 40% and 10% of ACTIVA BioACTIVE and MTA samples at the 30-day interval, respectively. These results agreed with Alzoubi et al., [43], who found that 80% of MTA specimens revealed no pulp calcifications and 20% had a single large pulp calcification after the 3rd month of follow-up. Also, these results coincided with Moretton et al., [50] who investigated the biocompatibility of MTA using both subcutaneous and intraosseous implantation in rats, assessing tissue responses at 15, 30, and 60 days post-implantation; it was concluded that MTA initially triggered intense reactions, including coagulative necrosis and moderate dystrophic calcification, which gradually lessened to predominantly moderate levels over time.

In this study, fibronectin and osteopontin were selected as immunohistochemical markers to assess the odontogenic differentiation potential of ACTIVA BioACTIVE and MTA. Fibronectin expression increased significantly from day 15 to day 30 in both groups, with a more pronounced elevation observed in the ACTIVA BioACTIVE group. This enhanced expression in ACTIVA-treated samples may be attributed to its sustained release of calcium ions, which are known to stimulate fibronectin gene expression in dental pulp cells, thereby supporting odontoblastic differentiation [51]. Additionally, the early localization of fibronectin at the site of newly formed dentin in the ACTIVA group suggests a more active role in initiating the differentiation and matrix organization process. These results coincided with Mizuno and Banzai., [52], who treated human dental pulp cells with high concentrations of calcium or magnesium ions for 24 h, then measured fibronectin gene expression by the quantitative PCR method; they reported that calcium ions stimulated fibronectin gene expression in a dose-dependent manner, whereas magnesium ions did not affect its expression. Also, these findings were in agreement with Eftimoska et al., [53] who assessed the effects of MTA and Biodentine on the expression of the glycoproteins fibronectin (FN) and tenascin (TN) (key regulators of dentinogenesis) and concluded that a noticeable immunoreactivity for fibronectin (FN) and tenascin (TN) was observed within the dentin bridge beneath both MTA and Biodentine at 8 days, and it persisted even 30 days after application, demonstrating induction of reparative dentinogenesis.

Osteopontin (an acidic glycoprotein) is a mineralization marker that was used as an immunohistochemical indicator in this study assessment since the increased expression of OPN in reparative dentin indicates the presence of odontoblast cells [54]. This was in agreement with Daltoé et al., [55] and Okasha et al., [56], who used the same mineralization marker at the 90-day assessment. In the present study, osteopontin (OPN) expression increased in both treatment groups over time, with a notably higher rise observed in the ACTIVA BioACTIVE group. This upregulation of OPN, a key marker of mineralization, suggests that ACTIVA possesses a strong potential to support early hard tissue bridge formation. The enhanced OPN expression is likely due to ACTIVA’s ability to attract fibroblasts and osteoblast-like cells, which are the primary stimulants to the gene expression of hard tissue-related proteins [57]. This concurred with Jun et al. [36], who revealed that the biomineralization capability of ACTIVA BioACTIVE was reported to be equivalent to that of other pulp capping materials, such as Dycal and Theracal, and gives matching quantities of (Ca) and (OH) ions. Moreover, these results agreed with Hakki et al., [58] who revealed that MTA stimulates the formation of mineralized tissue and promotes the expression of mRNA for proteins involved in mineralization (Bone sialoprotein (BSP), OCN, collagen type I(COL I), and osteopontin (OPN)), which are essential for cementum repair and regeneration, also the lowest two concentrations of MTA used (0.02 and 0.002 mg/mL) induced biomineralization of immortalized cementoblasts (OCCM) cells. Also, the present study findings were in line with Youssef et al., [59], who found that osteopontin (OPN) and dentin sialophosphoprotein (DSPP) gene expression was increased by MTA, Emdogain, Biodentine, and Ca(OH)_2,_ indicating osteogenic and odontogenic differentiation of dental pulp stem cells (DPSCs). Conversely, these findings were opposed to Abou ElReash et al. [14], who revealed that the extract of ACTIVA did not trigger the proliferation of human dental pulp stem cells (hDPSCs) in comparison to MTA-HP and iRoot-BP-Plus.

The limitations of the present study are short evaluation periods and small sample size; also, the non-blind nature of this study, due to the distinct handling characteristics of the tested materials, could introduce bias in the evaluation of the materials. A lack of clinical and radiographic evaluations to appraise overall clinical and radiographic success outcomes for ACTIVA BioACTIVE as a vital pulpotomy agent in primary teeth is also a limitation. Further studies should be done to investigate the physical characteristics of ACTIVA BioACTIVE base/liner. Also, more clinical studies with a longer follow-up period are desirable.

## Conclusion

The current study's findings concluded that:

 − ACTIVA BioACTIVE Base/Liner had equal mild and moderate pulp vascularity compared to severe pulp vascularity of MTA samples after 30 days.
 − The mean total scoring of pulp fibrosis in ACTIVA BioACTIVE Base/Liner was significantly greater than that of MTA group.
 − Non-significant statistical differences were found between ACTIVA BioACTIVE and MTA groups concerning odontoblastic layer organization and pulp stone presence at both intervals.
 − Fibronectin and OPN in both ACTIVA BioACTIVE and MTA groups were positive at the fibrotic and calcified areas.
 − ACTIVA BioACTIVE Base/Liner may be an appropriate alternate for MTA in primary teeth pulpotomy when the cost of treatment is a concern.

## Data Availability

Access to the datasets used and/or analysed in this study can be obtained from the corresponding author following an appropriate demand.
